# Supplementary material for: An 11 000-year-old giant muntjac subfossil from Northern Vietnam: implications for past and present populations
Source: R Soc Open Sci. 2019 Mar 13;6(3):181461. doi: 10.1098/rsos.181461 (PMC6458398; doi:10.1098/rsos.181461)

**Table S1.** AMS radiocarbon dates from the total excavated extent of the midden sequence from Hang Boi (HBC), Tràng An World Heritage Area, Northern Vietnam. Depths are shown relative to arbitrary site datum.

| **Site** | **No.** | **Context** | **Trench** | **Depth (m)** | **^14^C Age** | **±** | **AMS δ^13^C** | **Sample** | **calibrated age (2δ range) - years** |
| --- | --- | --- | --- | --- | --- | --- | --- | --- | --- |
| HBC | 2214 | 5115 | 1 | Sub-surf. | 9406 | 44 | -25 | UBA-10163 | 10541-10737 |
| HBC | - | 5005D | 1 | 498 | 9397 | 49 | -32 | UBA-8371 | 10508-10741 |
| **HBC** | **-** | **5105** | **1** | **497.6** | **9863** | **59** | **-29.8** | **UBA-8372** | **11178-11406** |
| HBC | 2116 | 5106 | 1 | 497.26 | 9900 | 50 | -21.4 | UBA-10165 | 11208-11409 |
| HBC | 2216 | 5109 | 1 | 497.06 | 9981 | 45 | -25.3 | UBA-10166 | 11260-11624 |
| HBC | - | 5019 | 1 | 496.3 | 10362 | 32 | -32 | UBA-8373 | 12057-12391 |
| HBC | 2192 | 5123 | 1 | 496.1 | 10497 | 45 | -30.6 | UBA-10167 | 12376-12583 |
| HBC | 2215 | 5127 | 1 | 495.62 | 10444 | 45 | -25.9 | UBA-10168 | 12123-12536 |
| HBC | 2366 | 5224 | 1 | 495.31 | 11741 | 43 | -32.7 | UBA-14887 | 13455-13645 |

**Table S2.** Details of comparative specimens and dental metrics (lower m2 and m3) collected during this study. Conventions follow Janis [42].

| **Collection** | **Specimen** | **TAXON** | **SLML** | **SLMW** | **TLML** | **TLMW** |
| --- | --- | --- | --- | --- | --- | --- |
| AMNH | M-56991 | *Muntiacus crinifrons* | 11.37 | 8.59 | 17.28 | 8.55 |
| NHMUK | 1.3.2.21 | *Muntiacus crinifrons* | 12.74 | 9.31 | 17.22 | 8.49 |
| NHMUK | 2010.484 | *Muntiacus vuquangensis* | 13.08 | 9.3 | 18.34 | 8.76 |
| AMNH | M-274588 | *Muntiacus vuquangensis* | 12.72 | 10.18 | 17.28 | 9.64 |
| AMNH | M-31599 | *Muntiacus muntjak curvostylis* | 12.09 | 7.33 | 15.92 | 7.93 |
| AMNH | M-31600 | *Muntiacus muntjak curvostylis* | 11.39 | 7.71 | 14.25 | 7.51 |
| AMNH | M-43052 | *Muntiacus muntjak vaginalis* | 11.06 | 8.45 | 15.72 | 8.83 |
| AMNH | M-43056 | *Muntiacus muntjak vaginalis* | 12.14 | 8.86 | 16.11 | 8.74 |
| AMNH | M-54594 | *Muntiacus muntjak vaginalis* | 11.25 | 8.68 | 15.19 | 8.32 |
| AMNH | M-54595 | *Muntiacus muntjak vaginalis* | 10.67 | 8.22 | 15.37 | 8.11 |
| AMNH | M-54596 | *Muntiacus muntjak vaginalis* | 13.59 | 8.32 | 14.97 | 8.66 |
| AMNH | M-54957 | *Muntiacus muntjak grandicornis* | 12.32 | 9.05 | 17.35 | 9.63 |
| AMNH | M-87604 | *Muntiacus muntjak annamensis* | 13.52 | 8.39 | 16.67 | 8.41 |
| AMNH | M-113480 | *Muntiacus muntjak vaginalis* | 11.21 | 8.92 | 16.29 | 9.24 |
| AMNH | M-114550 | *Muntiacus muntjak vaginalis* | 11.73 | 8.49 | 16.89 | 8.95 |
| AMNH | M-114553 | *Muntiacus muntjak vaginalis* | 12.08 | 9.41 | 17.47 | 9.05 |
| AMNH | M-114555 | *Muntiacus muntjak vaginalis* | 12.82 | 9.01 | 17.15 | 8.91 |
| AMNH | M-163635 | *Muntiacus muntjak vagnalis* | 11.15 | 9.12 | 16.6 | 9.17 |
| AMNH | M-163637 | *Muntiacus muntjak vagnalis* | 10.93 | 8.78 | 16.7 | 8.94 |
| AMNH | M-163638 | *Muntiacus muntjak vagnalis* | 12.39 | 8.46 | 16.08 | 7.64 |
| NHMUK | 701..a.a | *Muntiacus muntjak vagnalis* | 12.43 | 8.38 | 17.56 | 9.26 |
| NHMUK | 58.6.24.14 | *Muntiacus muntjak vagnalis* | 12.2 | 8.87 | 16.56 | 8.22 |
| NHMUK | 50.721 | *Muntiacus muntjak grandicornis* | 13.32 | 9.5 | 17.88 | 9.24 |
| NHMUK | 50.722 | *Muntiacus muntjak grandicornis* | 12.72 | 9.03 | 18.58 | 9.17 |
| NHMUK | 50.72 | *Muntiacus muntjak grandicornis* | 13.58 | 8.92 | 17.54 | 8.46 |
| NHMUK | 32.11.1.171 | *Muntiacus feae* | 13.08 | 8.1 | 15.8 | 8.1 |
| NHMUK | 24.1.6.2 | *Muntiacus feae* | 13.6 | 9.8 | 17.76 | 9.86 |
| AMNH | M-60767 | *Muntiacus muntjak* | 12.47 | 8.9 | 16.7 | 8.68 |
| OUMNH | ZC 20196 | *Muntiacus muntjak* | 12.1 | 7.74 | 16.28 | 8 |
| AMNH | M-43049 | *Muntiacus reevesi* | 10.03 | 6.56 | 13.44 | 6.65 |
| AMNH | M-45341 | *Muntiacus reevesi* | 8.99 | 7.01 | 12.62 | 6.59 |
| AMNH | M-45343 | *Muntiacus reevesi* | 9.25 | 6.94 | 12.06 | 6.48 |
| AMNH | M-45345 | *Muntiacus reevesi* | 9.81 | 6.89 | 11.58 | 6.57 |
| AMNH | M-45564 | *Muntiacus reevesi* | 8.92 | 7.45 | 13.25 | 7.13 |
| AMNH | M-45565 | *Muntiacus reevesi* | 9.5 | 6.67 | 13.44 | 6.7 |
| AMNH | M-45567 | *Muntiacus reevesi* | 9.47 | 6.88 | 13.41 | 6.49 |
| AMNH | M-45569 | *Muntiacus reevesi* | 9.41 | 6.75 | 13.31 | 6.7 |
| AMNH | M-45570 | *Muntiacus reevesi* | 9.3 | 6.87 | 12.92 | 6.81 |
| AMNH | M-55963 | *Muntiacus reevesi* | 10.68 | 8.13 | 15.49 | 8.06 |
| AMNH | M-56987 | *Muntiacus reevesi* | 9.51 | 7.18 | 13.88 | 6.8 |
| AMNH | M-56988 | *Muntiacus reevesi* | 8.58 | 6.13 | 13.53 | 5.98 |
| AMNH | M-56989 | *Muntiacus reevesi* | 9.37 | 6.67 | 13.4 | 6.24 |

**Table S2 ctd.**

| Collection | Specimen/Source | TAXON | **SLML** | **SLMW** | **TLML** | **TLMW** |
| --- | --- | --- | --- | --- | --- | --- |
| AMNH | M-57356 | *Muntiacus reevesi* | 8.94 | 6.63 | 12.62 | 6.53 |
| AMNH | M-59325 | *Muntiacus reevesi* | 10.75 | 7.35 | 14.25 | 6.76 |
| AMNH | M-59326 | *Muntiacus reevesi* | 10.23 | 6.87 | 12.36 | 6.98 |
| AMNH | M-59327 | *Muntiacus reevesi* | 9.91 | 6.76 | 14 | 6.82 |
| AMNH | M-60128 | *Muntiacus reevesi* | 9.44 | 6.93 | 13.44 | 6.89 |
| AMNH | M-60165 | *Muntiacus reevesi* | 8.73 | 6.42 | 12.55 | 6.51 |
| AMNH | M-60168 | *Muntiacus reevesi* | 9.71 | 7.17 | 13.45 | 7.04 |
| AMNH | M-60169 | *Muntiacus reevesi* | 10.04 | 6.75 | 13.54 | 6.54 |
| AMNH | M-84454 | *Muntiacus reevesi* | 9.78 | 6.78 | 13.07 | 6.42 |
| AMNH | M-84456 | *Muntiacus reevesi* | 10.01 | 7.33 | 12.87 | 7.23 |
| AMNH | M-84457 | *Muntiacus reevesi* | 9.66 | 7.37 | 14.13 | 7.07 |
| AMNH | M-89855 | *Muntiacus reevesi* | 11.06 | 7.75 | 13.85 | 7.96 |
| AMNH | M-184951 | *Muntiacus reevesi* | 9.13 | 7.31 | 13.52 | 6.46 |
| OUMNH | SR0483 | *Muntiacus reevesi* | 8.25 | 7.14 | 13.13 | 7.16 |
| NHMUK | 26.9.1.1 | *Elaphodus cephalophus* | 11.44 | 8.06 | 15.65 | 7.66 |
| NHMUK | 92.7.13.1 | *Elaphodus cephalophus* | 11.95 | 8.62 | 16.46 | 8.2 |
| NHMUK | 1.3.2.14 | *Elaphodus cephalophus* | 11.73 | 8.34 | 17.18 | 7.86 |
| NHMUK | 55.605 | *Elaphodus cephalophus* | 11.1 | 7.72 | 15.9 | 7.73 |
| NHMUK | 70.186 | *Elaphodus cephalophus* | 11.22 | 8.27 | 15.72 | 8.08 |
| NHMUK | 17.7.8.12 | *Hyelaphus porcinus* | 13.39 | 9.72 | 22.18 | 9.71 |
| NHMUK | 17.7.8.13 | *Hyelaphus porcinus* | 15.38 | 10.06 | 21.5 | 9.36 |
| NHMUK | 58.5.4.19 | *Hyelaphus porcinus* | 15.12 | 9.12 | 22.88 | 9.48 |
| NHMUK | 52.2.28.6 | *Hyelaphus porcinus* | 15.31 | 9.92 | 22.58 | 9.74 |
| UMZC | H16052 | *Hyelaphus porcinus* | 15.32 | 8.32 | erupt. | 7.32 |
| OUMNH | ZC 21654 | *Capricornis sumatraensis* | 18.42 | 11.63 | 26.16 | 11.62 |
| NHMUK | 83.324 | *Capreolus (capreolus) pygargus* | 12.66 | 9.9 | 17.93 | 8.92 |

**Figure S1.** Locations of measurements of specimen HBC-27587. Conventions follow Janis [42].


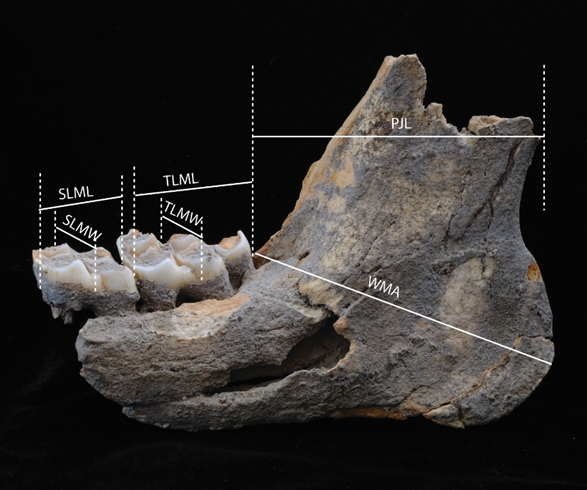

Supplement: Supplementary tables (2) and figures (1): Stimpson et al. [file rsos181461supp1.docx]
